# Nodular syphilitic scleritis masquerading as an ocular tumor

**DOI:** 10.1186/s12348-015-0040-5

**Published:** 2015-03-25

**Authors:** Sufiyan I Shaikh, Jyotirmay Biswas, Pukhraj Rishi

**Affiliations:** Shri Bhagwan Mahavir Vitreoretinal Services, 18, College Road, Sankara Nethralaya, Nungambakkam, Chennai, 600006, Tamil Nadu India; Uveitis & Ocular Pathology Department, Vision Research Foundation, 18, College Road, Nungambakkam, Chennai, 600006, Tamil Nadu India

**Keywords:** Syphilis, Scleritis, Tumor, Inflammation, Ocular masquerade

## Abstract

**Background:**

Scleritis may be the initial or only presenting feature of systemic, autoimmune, or infectious disorders. Corticosteroids are the mainstay of treatment for immune-mediated scleritis. However, steroids could prove detrimental when used to treat infectious scleritis. Hence, infectious causes of scleritis should be ruled out.

**Findings:**

A 47-year-old male from central India presented with swelling, pain, and redness in the left eye since 2 months. The patient was diagnosed elsewhere as having an extraocular extension of intraocular tumor and advised radiation brachytherapy for the same. Clinical examination revealed nodular scleritis in the left eye. The patient did not have any systemic illness or complaints suggestive of connective tissue disease. Laboratory investigations ruled out the same. However, Venereal Disease Research Laboratory (VDRL) test was positive. Rapid plasma reagin (RPR) test and *Treponema pallidum* hemagglutination assay (TPHA) were also positive, confirming the diagnosis of syphilis. Ultrabiomicroscopy (UBM) and ultrasound scan of the eye ruled out intraocular tumor. Treatment was initiated with benzathine penicillin 2.4 million units per week for 3 weeks to which the patient responded remarkably well.

**Conclusions:**

Although rare, syphilis can present as nodular scleritis masquerading as ocular tumor. Syphilis must be considered in the list of etiological diagnoses in patients presenting with nodular scleritis, and testing for this disease should be a part of routine investigation in patients with scleritis.

## Findings

### Introduction

Scleritis is a chronic inflammatory disease of the eye characterized by edema and cellular infiltration of the scleral and episcleral tissues, which can be vision threatening. Scleritis can be associated with autoimmune and less commonly infectious etiology [1,2]. Systemic autoimmune disorders, including rheumatoid arthritis, systemic lupus erythematosus, relapsing polychondritis, spondyloarthropathies, Wegener granulomatosis, polyarteritis nodosa, and giant cell arteritis, are often associated with it.

Scleritis may be the initial or only presenting clinical manifestation of many of systemic autoimmune or infectious disorders. Prompt and accurate diagnosis and systemic therapy can halt the relentless progression of both ocular and systemic processes, thus preventing destruction of the globe and prolonging survival. Infectious scleritis presents as an ulcerated or nonulcerated, inflamed scleral nodule, which may be associated with microabscess and areas of scleral necrosis [3]. It accounts for 5% to 10% of all cases of scleritis [4-6]. The mainstay of treatment for immune-mediated scleritis is corticosteroids which can prove to be of no use or on the contrary worsen the infectious scleritis; hence, infection should be ruled out.

Scleritis due to syphilis is a rare entity. To date, only few case reports have been described in literature [7,8]. We report a case of nodular syphilitic scleritis masquerading as an ocular tumor.

### Case report

A 47-year-old male came to our clinic with the chief complaints of swelling in the left eye since 2 months gradually increasing and associated with pain and redness. There was a swelling near the limbus temporally since the last 1.5 months, gradually increasing in size, with redness and intermittent mild pain. There was no history of trauma. There was no associated history of any systemic illness including fever or joint pains or recent onset of weight loss. The patient was on treatment with topical antibiotics and lubricating eye drops since 2 months. MRI done elsewhere showed a 10 × 7.5 mm well-defined mass lesion in the temporal aspect of the left eye with both intra- and extraocular components. The patient had been diagnosed as having an ocular tumor and advised plaque brachytherapy elsewhere and had been referred to our clinic for a second opinion.

**Figure 1:**
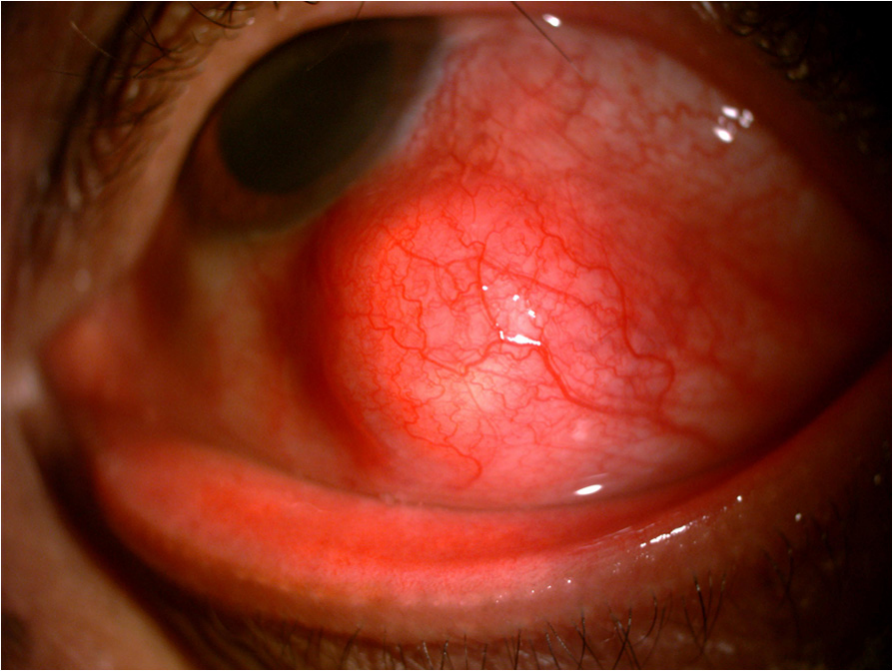
**Slit lamp picture of the left eye showing nodular scleritis.**

On examination, visual acuity was 6/5 in both eyes. Slit lamp examination of the left eye revealed a nodular, elevated mass arising from the sclera measuring 10 × 8 mm associated with congestion and mild tenderness suggestive of scleritis (Figure 1). There was no lymphadenopathy.

Fundus examination of the right eye was normal. Left eye fundus revealed an elevated mound in the inferotemporal quadrant corresponding externally to the mass lesion suggesting indentation effect by the mass. There was no evidence of any intraocular extension of the mass.

### Investigations

**Figure 2:**
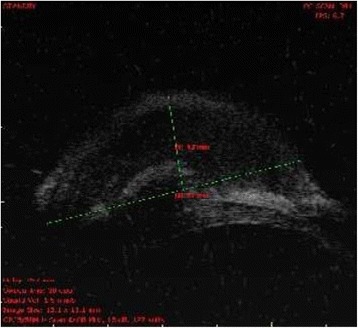
**UBM image of the left eye showing homogenous mass arising from the episcleral and scleral tissue.** No intraocular involvement is noted.

**Figure 3:**
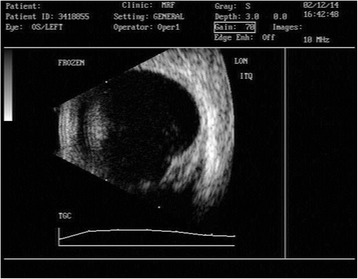
**Ultrasound B scan image of the left eye showing indentation by the scleral nodule.**

Ultrasound biomicroscopy showed the presence of homogenous mass in the inferotemporal quadrant arising from the sclera and episclera without any intraocular involvement (Figure 2). Ultrasound B scan image of the left eye showed indentation by the scleral nodule. There was no evident intraocular pathology on USG B scan (Figure 3).

**Figure 4:**
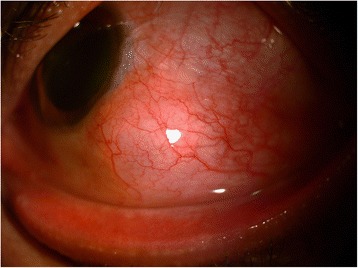
**Resolution of scleritis, status post 1 month of treatment with decrease in the size of scleral nodule.**

Systemic investigations revealed that the patient was positive for Venereal Disease Research Laboratory (VDRL) test. Anti-nuclear antibody (ANA) and rheumatoid arthritis factor (RA factor) tests were negative. Rapid plasma reagin (RPR) test and *Treponema pallidum* hemagglutination assay (TPHA) were positive, confirming the diagnosis of syphilis. After ruling out neurosyphilis by doing a CSF tap, treatment was initiated with benzathine penicillin 2.4 million units weekly regimen for 3 weeks. One-month follow-up revealed remarkable response with resolution of scleritis and decrease in the size of the scleral nodule, thus substantiating the diagnosis of syphilitic scleritis (Figure 4).

### Discussion

Syphilis is a sexually transmitted infection caused by the spirochete bacterium *Treponema pallidum* subspecies *pallidum*. The primary route of transmission is through sexual contact; it may also be transmitted from mother to fetus during pregnancy or at birth, resulting in congenital syphilis.

Syphilis has been divided classically into three stages [9,10]. The primary stage is characterized by the presence of chancre, a painless ulcerative lesion occurring at the site of inoculation which is usually the skin or mucous membrane. The chancre can be solitary or multiple and usually develops around 3 weeks after exposure to the organism (ranging from few days to 3 months). Primary lesions heal spontaneously in 2 to 8 weeks.

Secondary syphilis is characterized by the presence of rash which usually appears first on the trunk and then can spread to involve the entire body even affecting the palms and soles. There may be associated fever, sore throat, malaise, and lymphadenopathy. The symptoms from this stage resolve irrespective of treatment. Without treatment, secondary syphilis enters the latent stages and may progress to tertiary syphilis. CNS involvement can occur in about 25% of the patients with secondary syphilis. The patient is highly infectious in the secondary stage of syphilis.

The latent stage is characterized by an asymptomatic phase wherein there is a resolution of the previous symptoms but the patient still has asymptomatic bacteremia. The latent stage may last for many years. Latency can last 3 to 30 years and may or may not progress to the final or tertiary syphilis [11].

The tertiary stage refers to the final stage of the disease. It is characterized by the presence of granulomas (gummas) which can affect any organ in the body. Tertiary syphilis primarily affects the central nervous system and the cardiovascular system. Other organs affected include the liver, bone, joints, blood vessels, nerves, and eyes.

The term ‘quaternary’ syphilis has been described by some authors to refer to an aggressive form of tertiary syphilis occurring in immunocompromised individuals causing necrotizing meningoencephalitis [12], although there is not a common consensus on this terminology [13].

Syphilis has been referred to as the great imitator as it can mimic any disease [14]. This is true not only for the systemic manifestations but also for the ocular manifestation of this disease. Almost any ocular structure can be involved in syphilis, and it may mimic any ocular inflammatory disorder. The eye can be affected at any stage of syphilis [15]. However, ocular involvement is most frequently seen in the secondary and tertiary stages of syphilis [16].

Typically, the most common ophthalmic finding in ocular syphilis is panuveitis [17,18]. Other ocular manifestations include interstitial keratitis, intermediate uveitis, chorioretinitis, retinal vasculitis, retinitis, perineuritis, papillitis, retrobulbar neuritis, optic atrophy, optic nerve gumma, and various stroke syndromes [19-21]. Ocular syphilis may occur in both immunocompetent and immunocompromised hosts [19,22].

Episcleritis and scleritis caused by syphilis are rare entities. If present, they usually manifest as a feature of secondary or late syphilis [16]. Syphilitic episcleritis and scleritis are associated with lymphocytic infiltration along with vasculitis. These findings have been supported by anterior segment angiography [23]. Syphilitic scleritis should be differentiated from secondary intraocular inflammation caused by ciliary body gumma which in our case was normal as seen on ultrabiomicroscopic examination (UBM). Syphilis can masquerade any ocular pathology; hence, laboratory tests for syphilis should be a part of routine investigation for scleritis where the cause is not obvious, as scleritis can be the initial manifestation of syphilis [7].

Watson and Hayreh [7] in their review of 159 patients with episcleritis reported one case of nodular episcleritis caused by syphilis. Similarly, of 207 patients with scleritis, six (3%) had syphilis [7] and two with scleritis, who had ocular involvement as the initial manifestation of late syphilis.

Wilhelmus et al. [8] reported a case series of four patients, two with nodular episcleritis and two with scleritis, who had ocular involvement as the initial manifestation of late syphilis in 1987.

Recently published case series however do not report scleritis as a common manifestation of syphilis nor has it been reported as a common cause of infectious scleritis. Moradi et al. reported scleritis in 6.5% of the patients with ocular syphilis [24].

Riono et al. [3] in their case series of 55 patients found 11 patients (20%) to have infectious scleritis. The most common organism was pseudomonas in five patients. None of the patients had syphilis.

Sahu et al. [25] did not report any case of syphilitic scleritis in their study of 17 eyes with microbiologically proven infectious scleritis. Similarly, Jain et al. [6] in their study of 21 eyes with infectious scleritis found fungal infection as the most common cause. There were no cases with syphilis.

In conclusion, occurrence of scleritis in syphilis is rare. Etiological diagnosis of syphilis is important when scleritis is the sole manifestation of the disease. This ‘great imitator’ should always be considered in the list of differential diagnoses by ophthalmologists especially when the etiological diagnosis is difficult to determine.

### Consent

Written informed consent was obtained from the patient for the publication of this report and any accompanying images.

## Abbreviations

ANA: anti-nuclear antibody
RA factor: rheumatoid arthritis
RPR: rapid plasma reagin
TPHA: *Treponema pallidum* hemagglutination assay
UBM: ultrabiomicroscopy
VDRL: Venereal Disease Research Laboratory
